# Crystal structure of *catena*-poly[[aquadi-*n*-propyl­tin(IV)]-μ-oxalato]

**DOI:** 10.1107/S1600536814015372

**Published:** 2014-07-19

**Authors:** Martin Reichelt, Hans Reuter

**Affiliations:** a Institut für Chemie neuer Materialien, Universität Osnabrück, Barbarastrasse 7, D-49069 Osnabrück, Germany

**Keywords:** tin, oxalate, crystal structure

## Abstract

The title compound, [Sn(C_3_H_7_)_2_(H_2_O)(C_2_O_4_)]_*n*_, represents the first diorganotin(IV) oxalate hydrate to be structurally characterized. The tin(IV) atom of the one-dimensional coordination polymer is located on a twofold rotation axis and is coordinated by two chelating oxalate ligands with two slightly different Sn—O bond lengths of 2.290 (2) and 2.365 (2) Å, two symmetry-related *n*-propyl groups with a Sn—C bond lengths of 2.127 (3) Å, and a water mol­ecule with a Sn—O bond length of 2.262 (2) Å. The coordination polyhedron around the Sn^IV^ atom is a slightly distorted penta­gonal bipyramid with a nearly linear axis between the *trans-*oriented *n*-propyl groups [C—Sn—C = 176.8 (1)°]. The bond angles between the oxygen atoms of the equatorial plane range from 70.48 (6)° to 76.12 (8)°. A one-dimensional coordination polymer results from the less asymmetric bilateral coordination of the centrosymmetric oxalate anion, inter­nally reflected by two slightly different C—O bond lengths of 1.248 (3) and 1.254 (3) Å. The chains of the polymer propagate parallel to [001] and are held together by hydrogen bonds between water mol­ecules and oxalate anions of neighboring chains, leading to a two-dimensional network parallel to (100).

## Abstract 2

The Sn^IV^ atom in the title compound shows a slightly distorted penta­gonal–bipyramidal SnC_2_O_5_ coordination with the C atoms of the aliphatic chain in the axial positions.

## Chemical context   

In a previous paper (Reichelt & Reuter, 2014 ▶), we described the formation and structure of the first diorganotin(IV) oxalate (*Ox*), (*R*_2_Sn)*Ox* for *R* = *t*-butyl in the course of a systematical study on the reaction of diorganotin(IV) oxides with nitric acid (Reuter & Reichelt, 2014*a* ▶,*b* ▶). Applying similar reaction conditions to di-*n*-propyl­tin oxide resulted in the formation of the title compound as an unexpected side product. This diorganotin(IV) oxalate hydrate gives new insights into the structural chemistry of organotin(IV) oxalates.
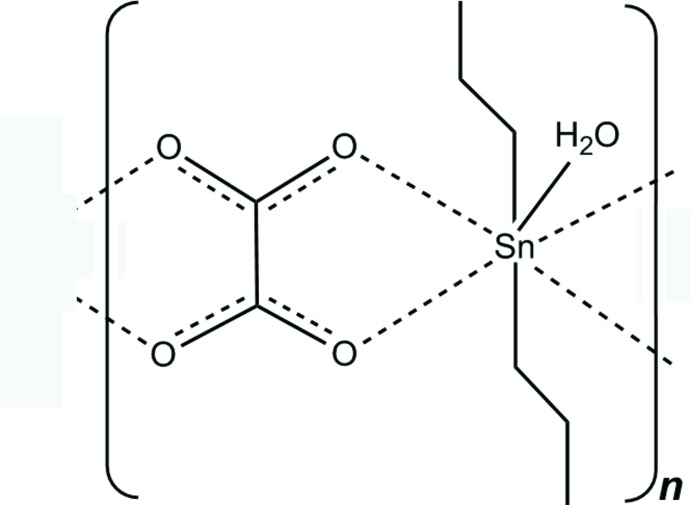


## Database survey   

Up to now, organotin(IV) oxalates were limited to a few representatives with general formula (*R*_3_Sn)_2_ *Ox, viz. R* = phenyl (Diop *et al.*, 2003 ▶); *R* = cyclo­hexyl (Ng *et al.*, 1994 ▶) and a Lewis-base-stabilized one with general formula [*R*_3_Sn(*LB*)]_2_ *Ox, viz. R* = methyl, *LB* = H_2_O (Diop *et al.*, 1997 ▶).

## Structural commentary   

**Figure 1:**
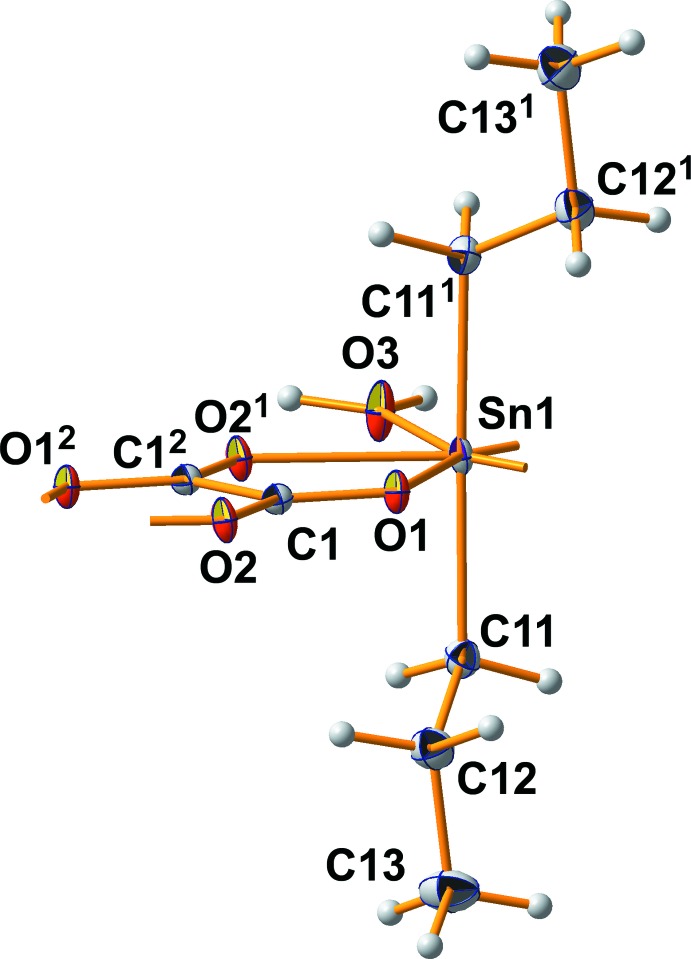
Ball-and-stick model of one formula unit in the crystal structure of the title compound with the atomic numbering scheme used. With exception of the H atoms, which are shown as spheres of arbitrary radius, all other atoms are drawn as displacement ellipsoids at the 50% probability level. [Symmetry codes: (1) 1 − *x*, *y*, ½ − *z*; (2) 1 − *x*, −*y*, 1 − *z*.]

The asymmetric unit of the title compound comprises one half of the formula unit (Fig. 1 ▶), consisting of an Sn^IV^ atom lying on a twofold rotation axis, a water mol­ecule with the O atom on the same rotation axis as the Sn atom, a bilateral chelating centrosymmetric oxalate anion and an *n*-propyl group attached to the Sn atom in general positions. Different from the unsubstituted *t*-butyl oxalate (Reichelt & Reuter, 2014 ▶), the Sn^IV^ atom is sevenfold coordinated by two *n*-propyl groups, four oxygen atoms of two symmetry-related oxalate anions and one water mol­ecule.

As a result of of symmetry, both Sn—C bond lengths are of equal length. At 2.127 (3) Å, they are considerably shorter than the Sn—C bond lengths of 2.186 (2) and 2.190 (2) Å in the di-*t*-butyl tin oxalate although the higher coordination number of the Sn atom in the hydrate compared with the Sn atom in the pure oxalate should result in longer bonds. This reflects the influence of the organic part (*n*-propyl *versus* *t*-but­yl) on Sn—C bond length, as already mentioned by Britton (2006 ▶). The *n*-propyl group itself is well ordered as can be deduced from the aniostropic displacement parameters as well as from the C—C bond lengths of 1.521 (3) and 1.522 (4) Å, which are in good agreement with the values reported by Allen *et al.* (1987 ▶) for *sp* ^3^-hybridized carbon atoms [1.513 (14) for –CH_2_—CH_3_, 1.524 (14) Å for –CH_2_—CH_2_–]. The corresponding bond angles are 117.0 (2) at C11 and 112.1 (2)° at C12. All in all, this group adopts a nearly staggered conformation with an Sn1—C11—C12—C13 torsion angle of −174.3 (2)°. Although both *n*-propyl groups attached to the Sn atom are related to each other by the twofold rotation axis, the bond angle is not exactly 180° because the Sn—C bond is not exactly perpendicular to this axis.

**Figure 2:**
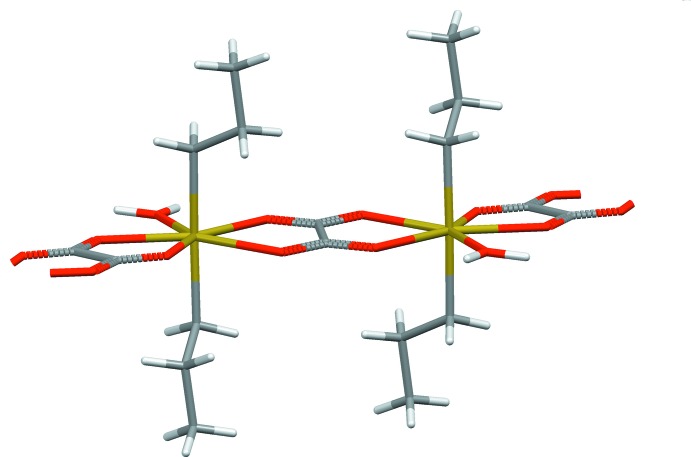
Stick-model showing a part of the one-dimensional coordination polymer. Colour code: Sn = bronze, O = red, C = dark grey, H = light grey.

The two symmetry-related oxalate anions coordinate side-on to the Sn atom with only slightly different Sn—O bond lengths [Sn1—O2 = 2.290 (2) Å and Sn1—O1 = 2.365 (2) Å]. This symmetrical coordination mode is in sharp contrast to the asymmetrical coordination mode of the oxalate anions in the anhydrous *t*-butyl compound [2.150 (1) to 2.4245 (1) Å] and is also reflected in C—O bond lengths which are much more closer to each other [C—O = 1.248 (3)/1.254 (3) Å, Δ = 0.006 Å] than in the *t*-butyl compound [1.242 (1)/1.269 (1) Å, Δ = 0.027 Å] as an expression of more delocalized C=O bonds. The oxalate ion itself is planar as it belongs to point group *C*_i_ and exhibits a C—C bond length of 1.549 (4) Å, [1.545 (3) Å], which is slightly longer than a normal bond between two *sp* ^2^-hybridized C atoms. From the bilateral, side-on coordination mode of the oxalate anion to the organotin moieties, a one-dimensional coordination polymer parallel to [001] results (Fig. 2 ▶).

**Figure 3:**
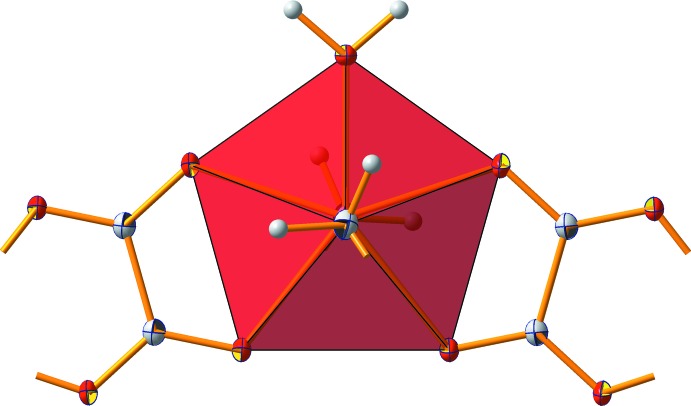
Schematic representation of the penta­gonal-bipyramidal coordination polyhedron around the Sn atom.

It is remarkable that the sevenfold coordination of the Sn atom corresponds to a penta­gonal bipyramid (Fig. 3 ▶). The axis formed by the two *n*-propyl groups is only slightly bent [176.8 (1)°] at the Sn atom. Only one [O1—Sn1—O1^i^ = 76.12 (8)°] of the five [O3—Sn1—O2^ii^/O2^iii^ = 71.60 (4)°; O1/O1^i^—Sn1—O2^iii^ = 70.48 (6)°; for symmetry codes see the Supporting information] bond angles between the O atoms of the equatorial plane deviates significantly from the ideal value of 72°. These structural features are caused (i) by the distance of the chelating oxalate anion to the Sn atom, (ii) by the symmetrical position of the water mol­ecule exactly between the two oxalate anions, and (iii) by a tilt of the plane of the oxalate anions relative to the least-squares plane through the atoms of the equatorial plane.

## Supra­molecular features   

**Figure 4:**
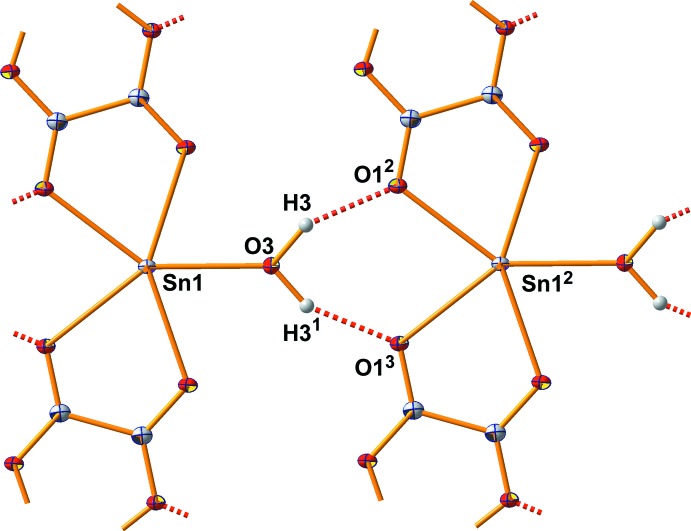
Part of the hydrogen-bonding (red dashed lines) system between adjacent chains of the one-dimensional coordination polymer. [Symmetry codes: (1) 1 − *x*, *y*, ½ − *z*; (2) *x*, 1 + *y*, *z*; (3) 1 − *x*, 1 + *y*, ½ − *z*.]

**Figure 5:**
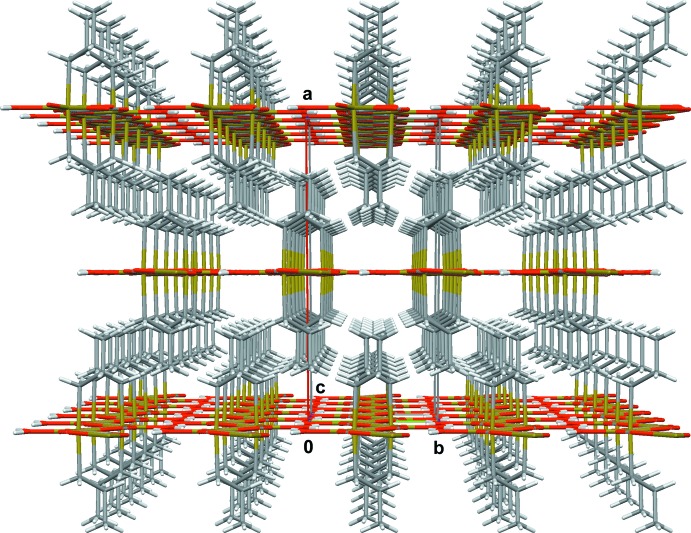
Perspective view of the crystal structure parallel to [001], looking down the chains of the one-dimensional coordination polymer.

In the solid state, this coordination polymer is stabilized by hydrogen bonds (Table 1 ▶) between the water mol­ecule of one chain as donor and the oxygen atom of the oxalate ion of neighboring chains as acceptor, and *vice versa*. As the plane of the water mol­ecule coincides with the propagation plane of the coordination polymer, an almost planar, two-dimensional linkage of the chains results (Fig. 4 ▶). These planes are staggered one above the other with the *n*-propyl groups of one plane protruding into the shell of *n*-propyl groups of the neighboring plane (Fig. 5 ▶).

## Synthesis and crystallization   

Single crystals of the title compound were obtained as side products during the reaction of di-*n*-propyl­tin(IV) oxide with a large excess of concentrated nitric acid in ethanol. In a typical experiment, a mixture of 0.32 g (1.45 mmol) ^*n*^Pr_2_SnO and 1.5 ml (21 mmol) HNO_3_ (Merck, 65%_wt_) in 5 ml ethanol was stirred at room temperature for several hours until a clear solution was obtained. Slow evaporation of solvents during some weeks resulted in the formation of colorless, block-shaped crystals of the title compound as well as crystals of an up-to-now unidentified reaction product. A suitable single crystal was selected under a polarization microscope and mounted on a 50 µm MicroMesh MiTeGen Micromount^TM^ using FROMBLIN Y perfluoro­polyether (LVAC 16/6, Aldrich).

## Refinement   

All hydrogen atoms could be localized in difference Fourier syntheses. Those of the *n*-propyl group were idealized and refined at calculated positions riding on the carbon atoms with C—H distances of 0.99 Å (–CH_2_–) and 0.98 Å (–CH_3_). Those of the water mol­ecule were refined with respect to a common O—H distance of 0.96 Å and an H—O—H bond angle of 104.5° before they were fixed and allowed to ride on the corresponding oxygen atom. For the hydrogen atoms of the *n*-propyl group, a common isotropic displacement parameter was refined as well as one common isotropic displacement parameter for the hydrogen atoms of the water mol­ecule. Experimental details are summarized in Table 2 ▶.

## Supplementary Material

Crystal structure: contains datablock(s) I. DOI: 10.1107/S1600536814015372/wm5030sup1.cif

Structure factors: contains datablock(s) I. DOI: 10.1107/S1600536814015372/wm5030Isup2.hkl

CCDC reference: 1011391

Additional supporting information:  crystallographic information; 3D view; checkCIF report

## Figures and Tables

**Table 1:** Hydrogen-bond geometry (Å, °)

*D*—H⋯*A*	*D*—H	H⋯*A*	*D*⋯*A*	*D*—H⋯*A*
O3—H3⋯O1^i^	0.96	1.87	2.741 (3)	149

Symmetry code: (i) 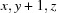.

**Table 2:** Experimental details

Crystal data	
Chemical formula	[Sn(C_3_H_7_)_2_(C_2_O_4_)(H_2_O)]
*M*_r_	310.90
Crystal system, space group	Monoclinic, *C*2/*c*
Temperature (K)	100
*a*, *b*, *c* (Å)	16.6490 (8), 6.4457 (3), 11.5438 (6)
β (°)	116.772 (2)
*V* (Å^3^)	1106.02 (9)
*Z*	4
Radiation type	Mo *K*α
μ (mm^−1^)	2.31
Crystal size (mm)	0.20 × 0.15 × 0.10

Data collection	
Diffractometer	Bruker APEXII CCD
Absorption correction	Multi-scan (*SADABS*; Bruker, 2009 ▶)
*T*_min_, *T*_max_	0.657, 0.811
No. of measured, independent and observed [*I* > 2σ(*I*)] reflections	19736, 1327, 1255
*R*_int_	0.062
(sin θ/λ)_max_ (Å^−1^)	0.660

Refinement	
*R*[*F* ^2^ > 2σ(*F* ^2^)], *wR*(*F* ^2^), *S*	0.024, 0.058, 1.11
No. of reflections	1327
No. of parameters	68
H-atom treatment	H-atom parameters constrained
Δρ_max_, Δρ_min_ (e Å^−3^)	1.49, −0.91

Computer programs: *APEX2* and *SAINT* (Bruker, 2009 ▶), *SHELXS97*, *SHELXL97* and *SHELXTL* (Sheldrick, 2008 ▶), *DIAMOND* (Brandenburg, 2006 ▶) and *Mercury* (Macrae *et al.*, 2008 ▶).

## supplementary crystallographic information

### Crystal data

**Table:**

[Sn(C_3_H_7_)_2_(C_2_O_4_)(H_2_O)]	*F*(000) = 616
*M**_r_* = 310.90	*D*_x_ = 1.867 Mg m^−^^3^
Monoclinic, *C*2/*c*	Mo *K*α radiation, λ = 0.71073 Å
Hall symbol: -C 2yc	Cell parameters from 9966 reflections
*a* = 16.6490 (8) Å	θ = 2.7–28.7°
*b* = 6.4457 (3) Å	µ = 2.31 mm^−^^1^
*c* = 11.5438 (6) Å	*T* = 100 K
β = 116.772 (2)°	Plate, colourless
*V* = 1106.02 (9) Å^3^	0.20 × 0.15 × 0.10 mm
*Z* = 4	

### Data collection

**Table:**

Bruker APEXII CCD diffractometer	1327 independent reflections
Radiation source: fine-focus sealed tube	1255 reflections with *I* > 2σ(*I*)
Graphite monochromator	*R*_int_ = 0.062
φ and ω scans	θ_max_ = 28.0°, θ_min_ = 2.7°
Absorption correction: multi-scan (*SADABS*; Bruker, 2009)	*h* = −20→21
*T*_min_ = 0.657, *T*_max_ = 0.811	*k* = −8→8
19736 measured reflections	*l* = −15→13

### Refinement

**Table:**

Refinement on *F*^2^	Primary atom site location: structure-invariant direct methods
Least-squares matrix: full	Secondary atom site location: difference Fourier map
*R*[*F*^2^ > 2σ(*F*^2^)] = 0.024	Hydrogen site location: inferred from neighbouring sites
*wR*(*F*^2^) = 0.058	H-atom parameters constrained
*S* = 1.11	*w* = 1/[σ^2^(*F*_o_^2^) + (0.0357*P*)^2^ + 0.058*P*] where *P* = (*F*_o_^2^ + 2*F*_c_^2^)/3
1327 reflections	(Δ/σ)_max_ < 0.001
68 parameters	Δρ_max_ = 1.49 e Å^−^^3^
0 restraints	Δρ_min_ = −0.91 e Å^−^^3^

### Special details

**Table:**

Geometry. All e.s.d.'s (except the e.s.d. in the dihedral angle between two l.s. planes) are estimated using the full covariance matrix. The cell e.s.d.'s are taken into account individually in the estimation of e.s.d.'s in distances, angles and torsion angles; correlations between e.s.d.'s in cell parameters are only used when they are defined by crystal symmetry. An approximate (isotropic) treatment of cell e.s.d.'s is used for estimating e.s.d.'s involving l.s. planes.
Refinement. Refinement of *F*^2^ against ALL reflections. The weighted *R*-factor *wR* and goodness of fit *S* are based on *F*^2^, conventional *R*-factors *R* are based on *F*, with *F* set to zero for negative *F*^2^. The threshold expression of *F*^2^ > σ(*F*^2^) is used only for calculating *R*-factors(gt) *etc*. and is not relevant to the choice of reflections for refinement. *R*-factors based on *F*^2^ are statistically about twice as large as those based on *F*, and *R*- factors based on ALL data will be even larger.

### Fractional atomic coordinates and isotropic or equivalent isotropic displacement parameters (Å^2^)

**Table:**

	*x*	*y*	*z*	*U*_iso_*/*U*_eq_	
Sn1	0.5000	0.13504 (3)	0.2500	0.01108 (10)	
C11	0.35701 (18)	0.1258 (3)	0.1538 (3)	0.0165 (5)	
H111	0.3343	0.2589	0.1703	0.028 (3)*	
H112	0.3376	0.1174	0.0593	0.028 (3)*	
C12	0.31172 (17)	−0.0495 (4)	0.1905 (3)	0.0210 (5)	
H121	0.3245	−0.0336	0.2825	0.028 (3)*	
H122	0.3374	−0.1836	0.1816	0.028 (3)*	
C13	0.21034 (18)	−0.0522 (5)	0.1060 (3)	0.0304 (7)	
H131	0.1845	0.0790	0.1163	0.028 (3)*	
H132	0.1839	−0.1675	0.1325	0.028 (3)*	
H133	0.1974	−0.0698	0.0150	0.028 (3)*	
O1	0.50631 (12)	−0.1538 (2)	0.38012 (17)	0.0131 (4)	
C1	0.50270 (17)	−0.1155 (3)	0.4836 (2)	0.0117 (5)	
O2	0.50337 (11)	−0.2472 (3)	0.56400 (15)	0.0141 (4)	
O3	0.5000	0.4860 (4)	0.2500	0.0203 (6)	
H3	0.5017	0.5767	0.3170	0.033 (8)*	

### Atomic displacement parameters (Å^2^)

**Table:**

	*U*^11^	*U*^22^	*U*^33^	*U*^12^	*U*^13^	*U*^23^
Sn1	0.01603 (15)	0.01051 (14)	0.00986 (14)	0.000	0.00864 (10)	0.000
C11	0.0170 (12)	0.0195 (14)	0.0133 (12)	0.0013 (9)	0.0071 (10)	0.0020 (9)
C12	0.0209 (13)	0.0223 (14)	0.0207 (13)	−0.0020 (11)	0.0101 (11)	0.0024 (11)
C13	0.0226 (14)	0.0369 (17)	0.0298 (16)	−0.0058 (13)	0.0101 (12)	0.0091 (13)
O1	0.0198 (9)	0.0118 (8)	0.0100 (9)	0.0014 (6)	0.0088 (7)	0.0005 (5)
C1	0.0104 (11)	0.0139 (12)	0.0106 (11)	0.0001 (9)	0.0046 (9)	−0.0002 (9)
O2	0.0222 (9)	0.0126 (9)	0.0121 (8)	0.0015 (7)	0.0119 (7)	0.0013 (6)
O3	0.0420 (16)	0.0096 (12)	0.0156 (12)	0.000	0.0185 (12)	0.000

### Geometric parameters (Å, º)

**Table:**

Sn1—C11	2.127 (3)	C12—H121	0.9900
Sn1—C11^i^	2.127 (3)	C12—H122	0.9900
Sn1—O3	2.262 (2)	C13—H131	0.9800
Sn1—O2^ii^	2.290 (2)	C13—H132	0.9800
Sn1—O2^iii^	2.290 (2)	C13—H133	0.9800
Sn1—O1	2.365 (2)	O1—C1	1.248 (3)
Sn1—O1^i^	2.365 (2)	C1—O2	1.254 (3)
C11—C12	1.521 (3)	C1—C1^ii^	1.549 (4)
C11—H111	0.9900	O2—Sn1^ii^	2.290 (2)
C11—H112	0.9900	O3—H3	0.9600
C12—C13	1.522 (4)		
			
C11—Sn1—C11^i^	176.8 (1)	Sn1—C11—H111	108.0
C11—Sn1—O3	91.60 (6)	C12—C11—H112	108.0
C11^i^—Sn1—O3	91.60 (6)	Sn1—C11—H112	108.0
C11—Sn1—O2^ii^	90.23 (8)	H111—C11—H112	107.3
C11^i^—Sn1—O2^ii^	90.78 (8)	C11—C12—C13	112.1 (2)
O3—Sn1—O2^ii^	71.60 (4)	C11—C12—H121	109.2
C11—Sn1—O2^iii^	90.78 (8)	C13—C12—H121	109.2
C11^i^—Sn1—O2^iii^	90.23 (8)	C11—C12—H122	109.2
O3—Sn1—O2^iii^	71.60 (4)	C13—C12—H122	109.2
O2^ii^—Sn1—O2^iii^	143.20 (8)	H121—C12—H122	107.9
C11—Sn1—O1	91.65 (8)	C12—C13—H131	109.5
C11^i^—Sn1—O1	85.83 (8)	C12—C13—H132	109.5
O3—Sn1—O1	141.94 (4)	H131—C13—H132	109.5
O2^ii^—Sn1—O1	70.48 (6)	C12—C13—H133	109.5
O2^iii^—Sn1—O1	146.23 (6)	H131—C13—H133	109.5
C11—Sn1—O1^i^	85.83 (8)	H132—C13—H133	109.5
C11^i^—Sn1—O1^i^	91.65 (8)	C1—O1—Sn1	116.4 (1)
O3—Sn1—O1^i^	141.94 (4)	O1—C1—O2	125.9 (2)
O2^ii^—Sn1—O1^i^	146.23 (6)	O1—C1—C1^ii^	117.2 (3)
O2^iii^—Sn1—O1^i^	70.48 (6)	O2—C1—C1^ii^	116.9 (3)
O1—Sn1—O1^i^	76.12 (8)	C1—O2—Sn1^ii^	119.0 (2)
C12—C11—Sn1	117.0 (2)	Sn1—O3—H3	127.5
C12—C11—H111	108.0		
			
Sn1—C11—C12—C13	−174.3 (2)		

Symmetry codes: (i) −*x*+1, *y*, −*z*+1/2; (ii) −*x*+1, −*y*, −*z*+1; (iii) *x*, −*y*, *z*−1/2.

### Hydrogen-bond geometry (Å, º)

**Table:**

*D*—H···*A*	*D*—H	H···*A*	*D*···*A*	*D*—H···*A*
O3—H3···O1^iv^	0.96	1.87	2.741 (3)	149

Symmetry code: (iv) *x*, *y*+1, *z*.
